# Whole-Genome Sequences of Nonencapsulated Haemophilus influenzae Strains Isolated in Italy

**DOI:** 10.1128/genomeA.00110-15

**Published:** 2015-03-26

**Authors:** Maria Giufrè, Matteo De Chiara, Stefano Censini, Silvia Guidotti, Giulia Torricelli, Gabriella De Angelis, Rita Cardines, Mariagrazia Pizza, Alessandro Muzzi, Marina Cerquetti, Marco Soriani

**Affiliations:** a Department of Infectious, Parasitic and Immune-mediated Diseases, Istituto Superiore di Sanità, Rome, Italy; b Novartis Vaccines, Siena, Italy

## Abstract

Haemophilus influenzae is an important human pathogen involved in invasive disease. Here, we report the whole-genome sequences of 11 nonencapsulated H. influenzae (ncHi) strains isolated from both invasive disease and healthy carriers in Italy. This genomic information will enrich our understanding of the molecular basis of ncHi pathogenesis.

## GENOME ANNOUNCEMENT

Haemophilus influenzae colonizes the human nasopharyngeal mucosa and has the ability to cause a variety of infections, ranging from respiratory to invasive diseases. After the widespread use of the H. influenzae type b (Hib) conjugate vaccines, most invasive H. influenzae infections are nowadays caused by nonencapsulated (ncHi) strains (1–3). Despite the well-known genetic heterogeneity of the ncHi population (4), we recently identified a group of closely related clones belonging to multilocus sequence type 103 (ST103), ST139, and ST145 and representing almost 25% of all invasive ncHi strains circulating in Italy (5). In this study, we determined the whole-genome sequences of 11 ncHi strains (Table 1). Of these, 7 strains belonged to the above-mentioned major clones (6 from invasive disease and 1 from a healthy carrier) and 4 strains (2 from invasive disease and 2 from healthy carriers) were included into uncommon STs (ST34 and ST105).

**TABLE 1:** Summary of genome sequencing for the 11 H. influenzae isolates in the present study

Isolate	Source	BioSample no.	ST*^a^*	Accession no.	Genome coverage (×)	Genome size (Mb)	No. of contigs	No. of genes
MiHi64	Nasopharyngeal carriage	SAMN03284980	34	JXMG00000000	53	1.98	109	1,986
Hi345	Blood	SAMN03284972	34	JXLY00000000	71	1.81	22	1,776
Hi359	Blood	SAMN03284973	103	JXLZ00000000	60	1.86	30	1,847
Hi378	CSF*^b^*	SAMN03284975	103	JXMB00000000	48	1.92	26	1,909
RMHi93	Oropharyngeal carriage	SAMN03284981	105	JXMH00000000	47	1.94	35	1,938
Hi322	CSF	SAMN03284971	105	JXLX00000000	83	1.91	33	1,925
MiHi270	Nasopharyngeal carriage	SAMN03284979	139	JXMF00000000	60	1.84	26	1,812
Hi361	Blood	SAMN03284974	139	JXMA00000000	50	1.84	21	1,805
Hi381	CSF	SAMN03284976	139	JXMC00000000	50	1.84	24	1,808
Hi394	Blood	SAMN03284977	145	JXMD00000000	58	1.90	31	1,898
Hi403	CSF	SAMN03284978	145	JXME00000000	69	1.87	31	1,863

^a^ ST, sequence type.

^b^ CSF, cerebrospinal fluid.

The genomic DNAs were sequenced using Illumina MiSeq (100-bp paired-end reads) technology (Illumina, San Diego, CA). The genome sequences were assembled *de novo* using Celera Assembler7 (6). The resulting coverage ranged from 47× to 83×, with an average of 59×. Genome annotation was performed using Glimmer3 (7) and RATT (8).

The results of genome sequencing are summarized in Table 1. Overall, the ncHi genomes consist of only chromosomal DNA, without any plasmids, with an average length of 1.88 Mb and 1,870 putative protein-coding genes.

In-depth characterization of the reported genomes, including comparative analysis with other publicly available H. influenzae genomes, is under way. The herein-announced 11 genome sequences will help our understanding of the different pathogenic potentials of ncHi invasive isolates, contributing to the identification of relevant molecular and virulence markers possibly associated with the major clones.

### Nucleotide sequence accession numbers.

The genome sequences of the 11 ncHi isolates have been deposited in GenBank with the accession numbers JXLX00000000 to JXLZ00000000 and JXMA00000000 to JXMH00000000. The versions described in this paper are the first versions, JXLX01000000 to JXLZ01000000 and JXMA01000000 to JXMH01000000, respectively.
